# Neonatal screening for spinal muscular atrophy: Report of a multicenter study in Brazil

**DOI:** 10.1590/1678-4685-GMB-2025-0159

**Published:** 2026-05-08

**Authors:** Alice Brinckmann Oliveira, Marina Hentschke-Lopes, Fernanda Bender Pasetto, Thaís de Almeida Rocha dos Anjos, Gleice Santos de Andrade, Maria Inês Andrade Sousa, Inamara da Silva Moraes, Franciele Barbosa Trapp, Ana Carolina Brusius-Facchin, Jonas Alex Morales Saute, Michele Michelin Becker, Vivian de Lima Spode Coutinho, Santiago Vilella-Arias, Vitor Ponci, Marcondes Cavalcante França, Marcial Francis Galera, Tatiana Amorim, Roberto Giugliani

**Affiliations:** 1 Universidade Federal do Rio Grande do Sul, Programa de Pós-Graduação em Genética e Biologia Molecular, Porto Alegre, Brazil.; 2 Hospital de Clinicas de Porto Alegre, Laboratório BioDiscovery, Porto Alegre, Brazil.; 3 Instituto de Genética para Todos (IGPT), Porto Alegre, RS, Brazil.; 4 Casa dos Raros, Porto Alegre, RS, Brazil.; 5 Instituto Nacional de Genética Médica Populacional (INAGEMP), Porto Alegre, RS, Brazil.; 6 Hospital Universitário Júlio Müller, Serviço de Referência em Triagem Neonatal do Mato Grosso, Cuiabá, MT, Brazil.; 7 Associação de Pais e Amigos dos Excepcionais (APAE), Salvador, Salvador, Brazil.; 8 Hospital de Clinicas de Porto Alegre, Serviço de Genético Médica, Brazil.; 9 Universidade Federal do Rio Grande do Sul, Departamento de Medicina Interna, Porto Alegre, RS, Brazil.; 10 Hospital de Clínicas de Porto Alegre, Unidade de Neurologia Infantil, Porto Alegre, RS, Brazil.; 11 Hospital Materno Infantil Presidente Vargas, Serviço de Referência em Triagem Neonatal do Rio Grande do Sul, Porto Alegre, RS, Brazil.; 12 Universidade Federal de Ciências da Saúde de Porto Alegre, Programa de Pós-Graduação em Ciências da Saúde, Porto Alegre, RS, Brazil.; 13 Novartis Biosciences SA, São Paulo, SP, Brazil.; 14 Universidade Estadual de Campinas, Departamento de Neurologia, Campinas, Brazil.; 15 Universidade Federal do Mato Grosso, Faculdade de Medicina, Cuiabá, MT, Brazil.; 16 Universidade do Estado da Bahia, Faculdade de Medicina, Salvador, BA, Brazil.; 17 DASA Genômica, São Paulo, SP, Brazil.

**Keywords:** Spinal Muscular Atrophy, Neonatal Screening, Brazil, 5q-SMA, *SMN1* gene

## Abstract

Spinal muscular atrophy (SMA) is considered one of the most common autosomal recessive disorders, with an estimated incidence of 1 in 10,000 live births. Testing for SMA has been recommended for inclusion in neonatal screening (NBS) panels since there are several therapies available and there is evidence of greater efficacy when introduced in the pre/early symptomatic phases. In the present study, dried blood spot samples collected by the Reference Services of Neonatal Screening of the Brazilian states of Rio Grande do Sul, Sao Paulo, Mato Grosso and Bahia to perform the routine NBS panel were also screened for 5q-SMA, using real-time PCR (SALSA MC002 technique). In this study, samples from 80,000 newborns were analyzed, enabling the identification of 7 5q-SMA cases, which were confirmed by multiplex ligation-dependent probe amplification (MLPA). Considering our findings, Brazil has an incidence of 5q-SMA of 1 in 11,428 live births. This work expands regional knowledge about the incidence of SMA and is fundamental for planning the implementation of screening for this condition in Brazil.

Spinal muscular atrophy (SMA) is a severe autosomal recessive neuromuscular disease characterized by the degeneration of motor neurons in the spinal cord and brainstem, leading to progressive muscle weakness and atrophy (Lunn and Wang, 2008; Mercuri et al., 2012). Globally, the incidence of SMA is estimated to be approximately 1 in 10,000 live births, with a carrier frequency of around 1 in 50 individuals (D’Amico et al., 2011).

The primary genetic cause of 5q-Spinal Muscular Atrophy (5q-SMA) is a homozygous deletion or pathogenic variants in the *SMN1* (survival motor neuron 1) gene, including point mutations or small insertions/deletions (indels), which result in insufficient production of the survival motor neuron (SMN) protein, essential for motor neuron maintenance and survival (Lefebvre et al., 1995; Bürglen et al., 1996). The *SMN1* and *SMN2* genes are highly homologous and located within a large inverted duplication on chromosome 5q13, which predisposes to rearrangements and deletions. Although they share more than 99 % sequence identity, a single nucleotide change - the c.840C>T substitution in exon 7 of *SMN2* - causes an alteration in pre-mRNA splicing, resulting in a truncated and largely non-functional SMN protein (Lefebvre *et al.*, 1995; Bürglen *et al.*, 1996). While the *SMN2* copy number does not serve as a diagnostic criterion, it is a major modifier of disease severity and a determinant for therapeutic decision-making, particularly in the context of public health and early treatment eligibility (Finkel et al., 2017).

The severity of the disease varies widely, with different types of SMA classified according to the age of onset of symptoms and the maximum motor function achieved (D’Amico et al., 2011; Mercuri et al., 2012). In the current therapeutic era, patients are also frequently categorized according to functional status as “non-sitters,” “sitters,” or “walkers,” reflecting the level of achieved motor milestones rather than solely the traditional type I-IV classification (Mercuri et al., 2022). Without intervention, SMA leads to significant motor impairment, respiratory difficulties, and reduced life expectancy, particularly in its most severe type (Prior et al., 2010).

In this context, newborn screening (NBS) emerges as a crucial strategy for the early identification of infants with SMA, allowing for therapeutic intervention before the onset of clinical symptoms or at an early stage of the disease, optimizing the impact of treatment (Dangouloff et al., 2021; Cooper et al., 2024). The concept of NBS, widely established with the success of screening for phenylketonuria (Guthrie and Susi, 1963), was gradually expanded to include other conditions, aiming at the prevention of infant morbidity and mortality.

In Brazil, after some isolated non-governmental initiatives, NBS had its first formal steps with the implementation of the early diagnosis program for congenital hypothyroidism and phenylketonuria in 1992 (Ministério da Saúde, 1992), later consolidating with the creation of the National Newborn Screening Program (PNTN) in 2001 (Ministério da Saúde, 2001). Law No. 14,154/2021 represented an important milestone for the PNTN, expanding the number of screened diseases, including SMA in its 5^th^ phase (Ministério da Saúde, 2021). The incorporation of SMA into the NBS panel in Brazil reflects the recognition of the importance of early diagnosis to improve the prognosis of these patients, aligning with the positive experiences observed in other countries, and other regions of our country, that have already implemented this screening (Cooper et al., 2024; Romanelli et al., 2024; Berzal-Serrano et al., 2025; Hegedűs et al., 2025; Servais and Moreno, 2025). In a continental country like Brazil, laboratories that already have structure/experience can collaborate with the Reference Services of Neonatal Screening (SRTN), acting as reference centers for states that do not yet have the capacity to implement NBS for SMA; benefiting patients in appropriate times as recommended in the PNTN. This paper reports the update of an NBS project for SMA in Brazil (Oliveira Netto et al., 2023), by expanding the knowledge on SMA incidence and enhancing the robustness of data by integrating samples from a wide range of regions.

The study commenced only after obtaining approval from the local ethics committee at each participating center. Prior to sample collection, families were informed about the inclusion of a SMA test in the routine newborn screening panel. They were also given the option to decline participation in the study and continue with the standard NBS panel, without the SMA test. The ethics committees approved the waiver of individual written informed consent through the provision of a parent information leaflet, following a procedure consistent with similar ongoing projects aimed at expanding newborn screening programs (Oliveira Netto et al., 2023). Phase 1 (March 2022-April 2023) and Phase 2 (November 2023-October 2024) were conducted consecutively as part of a multicenter incidence study, rather than a prospective pilot aligned with PNTN timelines.

Dried blood spot (DBS) samples from the conventional heel prick test of 80,000 newborns were collected in the primary health care units connected to the SRTN of the four Brazilian states: SRTN/RS - Rio Grande do Sul, SRTN/Unicamp - Sao Paulo, SRTN/UFMT - Mato Grosso and SRTN/APAE Salvador - Bahia. These samples were then shipped to a research laboratory at Hospital de Clinicas de Porto Alegre (HCPA) where they were processed and analyzed.

DNA was extracted from the DBS samples of all newborns included in the study, following the method described in SALSA MC002 SMA Newborn Screen kit (MRC-Holland) (Strunk et al., 2019). These DNA samples were analyzed using real-time qPCR (first-tier test) with the SALSA MC002 SMA Newborn Screen kit (MRC-Holland) on the QuantStudio 5 real-time PCR system (Applied Biosystems). This reaction utilizes a single primer pair to amplify a region of exon 7 that includes the c.840C>A polymorphism, which differentiates the *SMN1* gene from the *SMN2* gene. Following amplification, a fluorescent probe will hybridize to the PCR amplicons, and fluorescence will be measured during the generation of a melting curve (*SMN1* Tm 64 ºC and *SMN2* Tm 57 ºC). The absence of a specific peak for *SMN1* will indicate exon 7 deletion, as described by Strunk *et al.* (2019). Positive or potentially positive cases were further analyzed (second-tier test) using Multiplex Ligation-dependent Probe Amplification (MLPA) with the specific SALSA MLPA Probemix P060 SMA kit (MRC-Holland). These analyses were performed according to the manufacturer’s protocol to determine the copy number of *SMN2*. These results allow the identification of potentially affected SMA patients, who were invited to collect a new sample for confirmatory laboratory procedures.

This phase of the study lasted 11 months (November 2023 to October 2024) and had 40,000 DBS analyzed samples. The division by states was organized in such a way that 10,000 DBS samples collected at SRTN/RS, 639 DBS samples collected at SRTN/Unicamp, 20,000 collected at SRTN/UFMT and 9,361 collected at SRTN/APAE Salvador were sent to the research laboratory for the SMA screening. Combining the first and second phases of the study, 80,000 samples from newborns were screened for the disease. The first phase of the study lasted 13 months (March 2022 to April 2023).

Out of the 80,000 samples screened, a total of 79,992 presented negative results for SMA in the first-tier test. The samples that obtained a negative result presented melt peaks at 64 ºC (*SMN1*) and 56 ºC (*SMN2*), or only at 64 ºC. The absence of a melt peak corresponding to the *SMN2* gene has no clinical consequences and it occurs frequently. As far as we know, no newborns carrying a homozygous deletion of *SMN1* gene were missed within the samples screened in this study.

Seven samples obtained positive results for SMA in the first-tier test and had the result confirmed by the second-tier test. The samples that obtained suggestive results for SMA presented a melt peak at 56 ºC (*SMN2*) only. Thus, MLPA (second-tier test) was performed enabling the confirmation of deletion and determination of the number of copies of *SMN2* exon 7, which serves as a predictor of the SMA clinical type. In these seven samples the deletion of *SMN1* was confirmed. Among them, two samples presented 4 copies of *SMN2* exon 7, three samples presented 3 copies of *SMN2* exon 7 and the other two samples presented 1 copy of *SMN2* exon 7 (Table 1).

**Table 1:** Clinical information, genotype, and clinical type of patients positive for SMA in the NBS.

Sex	Age at sample collection¹	Age at sample analysis²	Age at confirmatory genetic diagnosis	Number of copies of SMN2	SRTN
Male	2 months	3 months	4 months	4 copies	SRTN/RS
Male	4 days	1.5 months	2 months	3 copies	SRTN/RS
Female	5 days	2.5 months	3 months	3 copies	SRTN/RS
Male	6 days	2.5 months	3 months	2 copies	SRTN/Unicamp
Male	6 days	1.5 months	2 months	3 copies	SRTN/MT
Male	6 days	6 months	6 months	4 copies	SRTN/RS
Female	7 days	1 month	2.5 months	2 copies	SRTN/MT

SRTN: Reference Services of Neonatal Screening; RS: Rio Grande do Sul; MT: Mato Grosso.

¹Age when DBS samples were collected for the conventional heel prick test. ²Age at which sample arrived at the research laboratory for SMA screening.

One sample presented a positive result only in the first-tier test, but the second-tier test did not confirm the deletion. Consequently, a false positive result was obtained in the first-tier test, demonstrating the importance of performing MLPA for diagnostic confirmation. Further investigation using other techniques, such as long-range PCR or next-generation sequencing, could help identify possible SNPs within probe-binding regions or other causes for the isolated false-positive result.

The results of this study enabled the obtention of updated population data on the estimated incidence of SMA in Brazil, based on the frequency of positive cases. We identified 7 patients with the homozygous deletion of *SMN1*, providing an incidence of 1 in 11,428 live births. Beyond that, this work demonstrates the feasibility of the screening for 5q-SMA using samples collected for the conventional NBS test and transporting them from diverse and distant regions, which is extremely important for the successful expansion of the PNTN. Having said that, we acknowledge the limitation related to sample transportation from distant states, which may have impacted the turnaround time for first-tier results. Delays between sample collection and analysis were mainly due to logistics and centralized laboratory processing in Porto Alegre. This reflects real-world challenges in sample transport across large geographical distances. The samples were, however, transported under controlled temperature and humidity conditions, maintaining DNA integrity.

In Brazil, the Federal District and the State of Minas Gerais have been carrying out screening for SMA since 2023 and 2024, respectively, within the PNTN (Secretaria da Saúde do Distrito Federal, 2023; NUPAD, 2024). In addition, in the State of São Paulo, the Jô Clemente Institute is also carrying out a pilot project for SMA screening, and already reported results in 192,000 newborns (Romanelli et al., 2024). 

Pilot studies and statewide programs in various countries and regions such as Australia, Belgium, Canada, China, Germany, Hungary, Italy, Japan, Latvia, Netherlands, Norway, Poland, Portugal, Russia, Spain, Taiwan, Turkey, Ukraine, United Kingdom and United States have demonstrated the feasibility and value of performing NBS for SMA (Velikanova et al., 2022; Celik et al., 2024; Cooper et al., 2024; Berzal-Serrano et al., 2025; Hegedűs et al., 2025). By early 2024, 33 countries had reported either piloting or implementing established NBS programs for SMA (Vrščaj et al., 2024).

While the clinical benefits of early NBS and treatment are clear, the costs associated with implementing NBS programs and providing lifelong disease modifying therapies are substantial (Shih et al., 2022; Zanoteli, 2024). Developing a well-organized program is considered fundamental not only for achieving favorable outcomes for affected children but also for managing healthcare costs (Becker et al., 2024; Albuquerque et al., 2025). The cost-effectiveness of newborn screening for SMA has been demonstrated in various countries (Velikanova et al., 2022; Weidlich et al., 2023; Ghetti et al., 2024; Hata et al., 2025). Factors influencing costs include the screening methodology, the need for confirmatory testing, and the long-term available therapies (Romanelli et al., 2021). For example, the methodology used in this study, SALSA MC002 assay, requires manual handling and visual interpretation. Although effective and cost-efficient for regional laboratories, large-scale national implementation would benefit from automation or high-throughput platforms to optimize workflow and reproducibility. Newborn screening for SMA, combined with early access to disease-modifying therapies, serves as a critical public health policy. Data from Australia demonstrate its effectiveness in significantly reducing the disease burden, improving outcomes by a factor of seven, including slowing the progression of comorbidities, lowering mortality rates, and decreasing the need for ventilation (Kariyawasam *et al.*, 2023). As reported by Woodcock et al. (2025), the cost of screening is lower than the cost of symptomatic treatment and, in addition to this, the current scenario in Brazil must be considered, which already has treatment as a public policy.

In conclusion, this report brings the final results of a multicenter incidence study encompassing both phases (Oliveira Netto et al., 2023; present study), aiming to estimate 5q-SMA incidence across Brazilian regions, through the screening of 80,000 newborns over 24 months. DBS samples were collected by SRTN/RS (45,000), SRTN/Unicamp (5,639), SRTN/UFMT (20,000) and SRTN/APAE Salvador (9,631) and sent to the research laboratory for analysis (Figure 1). The identification of 7 patients with homozygous deletion in *SMN1* pointed to an incidence of 1 in 11,428 newborns. Nonetheless, we recognize the relatively short 24-month collection period, which may influence incidence estimates. Long-term follow-up data over ≥5 years is recommended to provide greater confidence in the incidence data.

**Figure 1:**
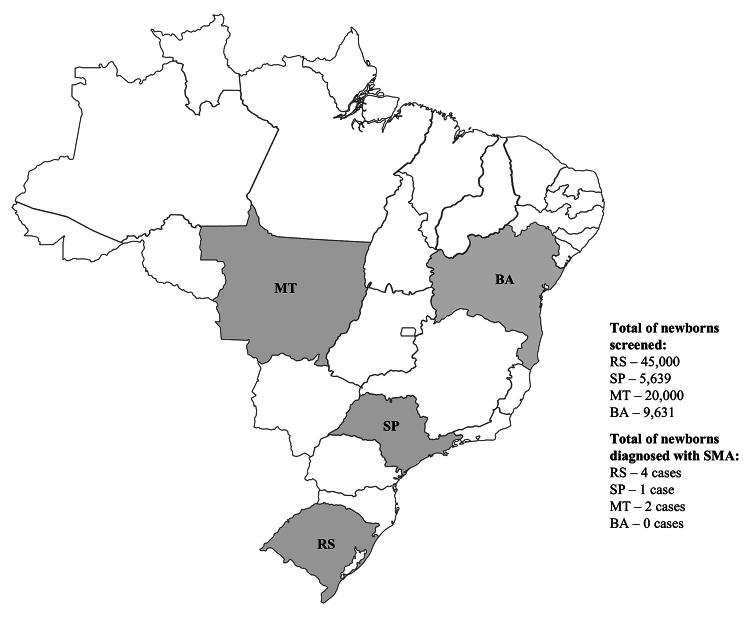
Map of Brazil indicating the regions included in the screening, with the number of newborns screened and positive cases found. BA, Bahia; MT, Mato Grosso do Sul; RS, Rio Grande do Sul; SP, São Paulo.

## Data Availability

The dataset that supports the results of this study is not publicly available
